# Physiological and Biological Responses to Short-Term Intermittent Hypobaric Hypoxia Exposure: From Sports and Mountain Medicine to New Biomedical Applications

**DOI:** 10.3389/fphys.2018.00814

**Published:** 2018-07-09

**Authors:** Ginés Viscor, Joan R. Torrella, Luisa Corral, Antoni Ricart, Casimiro Javierre, Teresa Pages, Josep L. Ventura

**Affiliations:** ^1^Physiology Section, Department of Cell Biology, Physiology and Immunology, Faculty of Biology, Universitat de Barcelona, Barcelona, Spain; ^2^Exercise Physiology Unit, Department of Physiological Sciences, Faculty of Medicine and Health Sciences, Universitat de Barcelona, L'Hospitalet de Llobregat, Barcelona, Spain

**Keywords:** intermittent hypoxia, erythropoiesis, angiogenesis, cardioprotection, bronchial asthma, neuroprotection, circulating stem cells, regenerative medicine

## Abstract

In recent years, the altitude acclimatization responses elicited by short-term intermittent exposure to hypoxia have been subject to renewed attention. The main goal of short-term intermittent hypobaric hypoxia exposure programs was originally to improve the aerobic capacity of athletes or to accelerate the altitude acclimatization response in alpinists, since such programs induce an increase in erythrocyte mass. Several model programs of intermittent exposure to hypoxia have presented efficiency with respect to this goal, without any of the inconveniences or negative consequences associated with permanent stays at moderate or high altitudes. Artificial intermittent exposure to normobaric hypoxia systems have seen a rapid rise in popularity among recreational and professional athletes, not only due to their unbeatable cost/efficiency ratio, but also because they help prevent common inconveniences associated with high-altitude stays such as social isolation, nutritional limitations, and other minor health and comfort-related annoyances. Today, intermittent exposure to hypobaric hypoxia is known to elicit other physiological response types in several organs and body systems. These responses range from alterations in the ventilatory pattern to modulation of the mitochondrial function. The central role played by hypoxia-inducible factor (HIF) in activating a signaling molecular cascade after hypoxia exposure is well known. Among these targets, several growth factors that upregulate the capillary bed by inducing angiogenesis and promoting oxidative metabolism merit special attention. Applying intermittent hypobaric hypoxia to promote the action of some molecules, such as angiogenic factors, could improve repair and recovery in many tissue types. This article uses a comprehensive approach to examine data obtained in recent years. We consider evidence collected from different tissues, including myocardial capillarization, skeletal muscle fiber types and fiber size changes induced by intermittent hypoxia exposure, and discuss the evidence that points to beneficial interventions in applied fields such as sport science. Short-term intermittent hypoxia may not only be useful for healthy people, but could also be considered a promising tool to be applied, with due caution, to some pathophysiological states.

## Intermittent hypoxia: concept and historical background

The term “intermittent hypoxia” is widely used and applies to a wide spectrum of situations that range from alpine expeditions to obstructive sleep apnea (OSA). However, in physiological terms, there are often few similarities between these conditions (Viscor et al., 2014). Although the same physiological responses are elicited by the same sensors and signaling pathways, the varied intensity and duration of the hypoxia switches different mechanisms on and off at different times, thus making the final physiological changes induced in the whole organism highly variable. In addition, a high variability in human tolerance to hypoxia has been reported, and it is now known to vary throughout the lives of individuals (Canouï-Poitrine et al., 2014; Richalet and Lhuissier, 2015). As a consequence, specialists have engaged in an interesting discussion about how to measure and define hypoxic “dosage” (Serebrovskaya et al., 2008; Garvican-Lewis et al., 2016). In general, three different types of intermittent hypoxia can be considered:

- Episodic intermittent hypoxia, which consists of successive short episodes of hypoxia with variable intensity. This may be present in permanent situations such as OSA, in transitory situations such as surgical ischemia-reperfusion (to one or several organs) and in some sporting activities such as parachuting and extreme skiing, in which the subject may not even notice the hypoxia.
- Intervallic intermittent hypoxia, which is characterized by long periods of normoxia interspersed with periods of hypoxia, as seen in frequent alpine expeditions and trekking at altitude, mountain rescue teams, regular intercontinental commercial flight crews (when in flight, commercial airplane cabins are usually regulated at pressures equivalent to a moderate altitude of about 2,000 m above sea level), and even astronauts at the International Space Station or future extraplanetary missions.
- Chronic intermittent hypoxia, which affects individuals who work under shift systems characterized by work at moderate or high altitudes alternating with periods of rest at sea level. This last model is common in the Andes region and in the Central Asian mining industry, but is also found in contractors for astronomical observatories, the military and customs, and police and border control personnel in many high-elevation countries.

The concept of “hypoxic training” was coined during the 1930s in the academic environment of the former Soviet Union, and was considered a useful therapeutic tool after it was shown to have beneficial effects on a number of different pathologies, even though the mechanisms involved in these favorable effects were unclear (Agadzhanyan and Torshin, 1986; Serebrovskaya, 2002). For instance, it was reported to have a beneficial effect on hypertension and cardiovascular diseases (Aleshin et al., 1993). Later, new experimental studies corroborated some of these findings and provided a fresh insight through enhanced plasma lipid profiles (Tin'kov and Aksenov, 2002). Nowadays in former Soviet States, hypoxic training is systematically applied as a non-pharmacological strategy for treating a wide range of alterations, including chronic lung disease, bronchial asthma, hypertension, diabetes mellitus, Parkinson's disease, emotional disorders and radiation toxicity, and for the prophylactic treatment of some occupational diseases (Ge et al., 1994; Xi and Serebrovskaya, 2012). In Western countries, intermittent hypoxia exposure programs were first applied in the field of sports medicine to improve aerobic capacity and for pre-acclimatization to altitude (Richalet et al., 1992; Levine and Stray-Gundersen, 1997; Rodríguez et al., 1999; Stray-Gundersen and Levine, 1999; Casas M. et al., 2000; Ricart et al., 2000).

Our group took an in-depth look at the physiological responses to intermittent exposure to hypobaric hypoxia (IEHH) in hypobaric (low barometric pressure) chambers. A detailed study of the precise mechanisms that underlie these adaptive responses (erythropoiesis, angiogenesis and the release of circulating stem cells) in humans and in an experimental rodent model encouraged us to explore the possibilities of applying IEHH programs with biomedical and therapeutic purposes (Panisello et al., 2007, 2008; Esteva et al., 2009; Viscor et al., 2009; Corral et al., 2014b). Thus, we recently demonstrated the efficacy of applying IEHH programs (passive exposure only or combined with exercise protocols) in the recovery of a range of injuries (Corral et al., 2014a; Núñez-Espinosa et al., 2014; Rizo-Roca et al., 2017a,b). These results are consistent with the new paradigm that proposes biphasic effects in the response to hypoxia (hormesis); that is, its harmful or beneficial effects depend on the frequency and severity of the hypoxic challenge to the organism or tissue in question (Navarrete-Opazo and Mitchell, 2014).

## Biological effects of intermittent hypoxia exposure

The molecular mechanisms involved in the response to hypoxia at the cellular level are relatively well understood (Fábián et al., 2016; Bhattarai et al., 2017; Koyasu et al., 2017). However, the complexity of the interactions between the divergent signaling pathways and the time frame of the various processes on different tissues and organs, together with the significant individual variability in humans' susceptibility to hypoxia (Rathat et al., 1992), pose formidable challenges to researching the potential benefits of regular programs involving exposure to real or simulated altitudes. This paper presents a series of examples of how intermittent exposure to hypoxia can benefit certain patients, although it is not intended to be an exhaustive list. Obviously, these programs must always be applied with due caution and under rigorous clinical controls, as with any pharmacological treatment.

### Erythropoiesis

Table 1 lists several representative studies of the favorable effects of intermittent hypoxia exposure by increasing erythropoiesis. A detailed report on the rate of erythropoietin (EPO) formation and plasma lifetime in humans in response to acute hypobaric hypoxia exposure in a hypobaric chamber was first provided by the group led by Christian Bauer in Zurich (Eckardt et al., 1989). Later, the pivotal role of hypoxia-inducible factor 1 (HIF-1) in the transcriptional response of EPO to hypoxia was also described (Wang and Semenza, 1993). These seminal works and many subsequent studies sparked an interest in applying IEHH programs to increase erythrocyte mass as a way of improving the aerobic capacity of athletes. Recombinant human erythropoietin (rHuEPO) opened up a new chapter in the correction of uremic anemia due to chronic renal failure (Egrie et al., 1986; London et al., 1989; Najean et al., 1989), but also led to unethical use in sports medicine. The celebration of the 1968 Summer Olympics held in Mexico City (2,250 m above sea level) aroused interest in studying the effects of altitude on human performance. The reduced performance in sporting events with a high aerobic component was evident, while participants in competitions with a clear anaerobic character saw no decline in their performance, and some even beat their records (Di Prampero et al., 1970). As a consequence, stays at high altitude and other artificial hypoxia exposure strategies in athletes were subject to intense study; a wide range of strategies, from permanent stays at moderate or high geographic altitudes (Antezana et al., 1994; Richalet et al., 1994) to different patterns of intermittent exposure (Levine and Stray-Gundersen, 1997; Chapman et al., 1998; Rodríguez et al., 1999; Casas M. et al., 2000; Karlsen et al., 2001; Stray-Gundersen et al., 2001; Ge et al., 2002; Lundby et al., 2007; Richalet and Gore, 2008) were examined. Noticeable differences in protocols and hypoxia exposure methods led to intense debate on the usefulness of intermittent hypoxia exposure for elite athletes, given that hypoxic dose and interindividual variability represent two of the main constraints (Chapman et al., 1998; Casas H. et al., 2000; Julian et al., 2004; Gore et al., 2006; Wilber et al., 2007; Truijens et al., 2008; Rodríguez et al., 2015). In parallel, there was rising interest in understanding the non-erythropoietic effects of EPO. The discovery of multi-tissue erythropoietin receptor expression provided an insight into erythropoietin (EPO) activity that went beyond its role in the regulation of red blood cell production, including a key role in cardioprotection, brain development and neuroprotection, through a coordinated response against tissue oxygen shortage (Noguchi et al., 2007; Arcasoy, 2008; Burger et al., 2009; Chateauvieux et al., 2011; Jia et al., 2012; Zhang Y. et al., 2014b), thus contributing to ischemic preconditioning, an interesting and important property of organ survival that could also prove very useful for new biomedical applications.

**Table 1:** Examples of the effects of intermittent hypoxia exposure on erythropoiesis.

**Subjects**	**Hypoxia time**	**Hypoxia method**	**Hypoxia dosage**	**Outcome**	**References**
Healthy men (*n* = 6)	5.5 h	HC	3,000–4,000 m	↑EPO	Eckardt et al., 1989
Alpinists (*n* = 10; 4 w + 6 m)	10 d	Altitude	6,542 m	↑Hct ↑[Hb]	Richalet et al., 1994
Competitive (*n* = 13; 4 w + 9 m)	4 h TL/d for 4 wk	Altitude (LH-TL) Altitude (LH-TH)	1,250/2,500 m	↑Hct ↑[Hb] ↑RBC_mass_	Levine and Stray-Gundersen, 1997
Competitive (*n* = 39; 12 w + 27 m)	30 h (14 d)	Altitude (LH-TL) Altitude (LH-TH)	1,200–1,400 m/2,500–3,000 m	↑EPO	Chapman et al., 1998
Alpinists (*n* = 17; 3 w + 14 m)	3–5 h/d for 9 d	HC	4,000–5,500 m	↑Hct ↑[Hb] ↑RBC	Rodríguez et al., 1999
Elite alpinists (*n* = 6; 1 w + 5 m) Alpinists (*n* = 17; 3 w + 14 m) Novice men (*n* = 8)	3–5 h/d for 17 d 3–5 h/d for 9 d 1.5 h/d × 3 d/wk for 3 wk	HC	4,000–5,500 m	↑Hct ↑[Hb] ↑RBC (all protocols)	Casas H. et al., 2000
Elite runners (*n* = 26; 9 w + 17 m)	4 wk (4 h TL/d)	Altitude LH-TL	1,225/2,500 m	↑EPO ↑Hct ↑[Hb]	Stray-Gundersen et al., 2001
Healthy people (*n* = 48; 16 w + 32 m)	24 h	HC	1,780m 2,085m 2,454m 2,800m	↑EPO (6 h) ↓EPO (24 h) ↑EPO (6 h) ↓EPO (24 h) ↑EPO (6 h) ↑EPO (24 h ↑EPO (6 h) ↑EPO (24 h)	Ge et al., 2002
Competitive (*n* = 17; 3 w + 14 m)	(IHT 5:5) 70 min/d (5 d/wk) for 4 wk	NH	FiO_2_ = 0.12 (≈4,400 m) FiO_2_ = 0.11 (≈5,200 m) FiO_2_ = 0.10 (≈5,800 m) FiO_2_ = 0.10 (≈5,800 m)	No changes	Julian et al., 2004
Competitive (*n* = 23; 12 w + 11 m)	3 h/d × 5 d/wk for 4 wk	HC	4,000–5,500 m	↑EPO (3 h after)	Gore et al., 2006
Competitive (*n* = 87; 28 w + 59 m)	4 wk (≥22 h) 4 wk (12–16 h)	Altitude HC	2,000–2,500 m 2,500–3,000 m	↑EPO	Wilber et al., 2007
Elite athletes (*n* = 41) XC Skiers (*n* = 11) Swimmers (*n* = 18) Runners (*n* = 12)	18 nights 13 nights 18 nights	NH and Altitude (LH-TL)	1,200/ /2,500–3,500 m /2,500–3,000 m /2,500–3,000 m	↑[Hb] ↑Hct ↑[Hb] ↑[Hb]	Richalet and Gore, 2008
Trained rats	4 h/d × 5 d/wk for 2 wk	HC	4,000 m	↑[Hb]↑Hct ↑RBC	Núñez-Espinosa et al., 2014
Male trained triathletes (*n* = 18) -Normoxia (*n* = 9) -Hypoxia (*n* = 9)	1 h/d × 2 d/wk for 7 wk	NH	FiO_2_ = 0.145-0.15 (≈2,800–2,500 m)	↑[Hb] ↑RBC	Ramos-Campo et al., 2015
Elite swimmers (*n* = 54; 30w+24m)	3 or 4 wk	Altitude LH-TH vs. LL-TL vs. LH-TH+TL	690/2,320 m	↑tHb_mass_ for LH-TH and LH-TH+TL	Rodríguez et al., 2015
Male well-trained triathletes (*n* = 28) -HH (*n* = 11) -NH (*n* = 10) -Normoxia (*n* = 7)	230 h for 18 d 238 h for 18 d	Altitude (LH-TL) NH (LH-TL)	<1,200/2,250m 1,150 m/(≈2,250 m)	↑Hb_mass_ (similar in NH & HH)	Hauser et al., 2016

*Altitude, geographic altitude; EPO, serum or plasma erythropietin levels; FiO_2_, Fraction of inspired oxygen; [Hb], Blood hemoglobin concentration; Hb_mass_, whole body hemoglobin mass; HC, Hypobaric Chamber; Hct, Hematocrit; IHT(5:5), intermittent hypoxic training, alternating 5 min hypoxia with 5 min normoxia along the session; LH-TH, Living High-Training High; LH-TL, Living High-Training Low; NH, normobaric hypoxia; RBC, red blood cell count; RBC_mass_, whole body erythrocytic mass*.

### Angiogenesis and muscle capillarization

In Table 2, some examples of the effects of intermittent hypoxia exposure on angiogenesis, vascular remodeling, muscle capillarization and hypertension are presented. In addition to its erythropoietic role, HIF-1 is the main mediator of angiogenesis in response to hypoxic conditions (Rey and Semenza, 2010) and has been considered a potential therapeutic target in many diseases. Two different strategies have been applied: HIF-1 upregulation for ischemic diseases (Li et al., 2014) and HIF-1 inhibition for cancer and endometriosis (Zhou et al., 2012; Bhattarai et al., 2017). Multiple angiogenic factors have been tested in the past; however, therapies that use only one proangiogenic agent to elicit angiogenesis were shown to be insufficient (Hirota and Semenza, 2006). Therefore, the addition of non-pharmacological treatments based on hypoxia-induced angiogenesis may be a successful strategy (Zimna and Kurpisz, 2015).

**Table 2:** Examples of the effects of intermittent hypoxia exposure on angiogenesis, vascular remodeling, muscle capillarization, and hypertension.

**Subjects**	**Time of hypoxia**	**Hypoxia method**	**Hypoxia dosage**	**Outcome**	**References**
Healthy (*n* = 32 m) 14 normoxic (SL) 18 hypoxic (≈2,500 m)	3 cycling/wk for 4 wk	NH	FiO_2_ = 0.16 (≈2,500 m)	↑Lipid peroxidation during hipoxia	Bailey et al., 2001
Males (*n* = 16) Double blind groups 8 healthy 8 prior myocardial infarction	IHT 3–4 × (3–5:3) 15 sessions in 3 wk	NH	FiO_2_ = 0.14–0.10 (≈3,500–≈5,800 m)	↑Aerobic capacity ↑exercise tolerance (without differences in patients)	Burtscher et al., 2004
Sedentary male rats	4 h/d, 5 d/wk for 22 d	HC	5,000 m	↑Capillary density ↓Diffusion distances for slow fibres	Panisello et al., 2008
Overweight to obese subjects group (*n* = 24)	4 wk training under hypoxia at 65% VO_2_max	NH	FiO_2_ = 0.15 (≈2,760 m)	Better physical fitness, metabolic risk markers, and body composition	Wiesner et al., 2010
Male Wistar rats (hyperlipidemia induced by 8 wk high-fat diet)	3 × (10 s:10 s) ischemia/reperfusion preceding 180 min reperfusion	Ischemic postconditioning	Ischemia: 30 min LAD occlusion followed by 180 min of reperfusion	Up-regulation of HIF-1α can be cardioprotective	Li et al., 2014

*FiO_2_, Fraction of inspired oxygen; HC, Hypobaric Chamber; HIF, hypoxia inducible factor; NH, Normobaric hypoxia; IHT, Intermittent hypoxic training alternating hypoxia with normoxia along the session; LAD, left anterior descending coronary artery; VO_2_max, maximal oxygen consumption capacity*.

The physiological response elicited by hypoxia at moderate altitude exposure (1,800–3,000 m) is low, but increases when combined with exercise, with additional specific responses that are not observed when similar exercise levels are carried out in normoxia (Bärtsch et al., 2008). Moreover, even greater adaptations are obtained when the hypoxic intervention is accompanied by high-intensity training (Faiss et al., 2013; Sanchez and Borrani, 2018).

Although altitude is generally associated with increased health risks in most patients and elderly individuals, several studies have reported therapeutic benefits associated with exercising in mild hypoxia in a variety of alterations (Bailey et al., 2001; Burtscher et al., 2004; Wiesner et al., 2010). As exercising in moderate hypoxia seems to play a valuable role as an additional “therapeutic strategy,” albeit one with both benefits and risks, new insights on this paradigm are now increasing. Some critical analysis and guidelines for hypertensive, obese and elderly individuals have recently been proposed (Millet et al., 2016). These concluded that intermittent hypoxic training seems to be well tolerated by most patients, in a similar way to healthy individuals, and that hypoxia and exercise may have additive or synergistic effects, probably mediated by several factors, including nitric oxide, angiogenesis and “altitude anorexia,” thereby paving the way for researchers to identify the optimal individual combination of exercise and hypoxia.

### Cardiac remodeling

Aerobic exercise activities have traditionally been widely recommended for preventing disease and promoting health. Today, resistance training is usually included in physical activity counseling, even for older adults and people with a range of cardiac conditions (Haskell et al., 2007; Nelson et al., 2007; Heuschmann et al., 2010), and there is solid evidence of different echocardiographic repercussions (Kenney et al., 2012). The cardioprotective effects of chronic intermittent hypoxia have been extensively studied, and their positive effect has shown to be related to preservation of mitochondrial function and inhibition of potassium channels sensitive to ATP (mitoKATP) present in the sarcoplasmic and mitochondrial membranes (Ostádal et al., 1989; Asemu et al., 1999, 2000; Chouabe et al., 2004; Ostadal and Kolar, 2007). Additional myocardial remodeling data were reported by our group using a model of IEHH (Panisello et al., 2007). Both chronic and intermittent exposure models supported the potential beneficial effects of acute exposure in coronary patients reported in pioneering studies conducted by Peruvian cardiologist Emilio Marticorena (Marticorena, 1993; Marticorena et al., 2001; Reynafarje and Marticorena, 2002; Valle et al., 2006). In rodents, it has been demonstrated that endurance exercise training and IEHH modulate cardiac mitochondria to a protective phenotype characterized by decreased induction of mitochondrial permeability transition pore and apoptotic signaling (Magalhães et al., 2013, 2014). However, there are no studies on humans on cardiac remodeling that combine hypoxia and exercise, other than those dealing with OSA models. There have been extensive studies on patients suffering from this syndrome, and several pieces of evidence may be found in the literature: the progression and reversibility of atrial remodeling following stretch release may help prevent atrial fibrillation (Thanigaimani et al., 2017), the important prognostic information of right-sided heart dysfunction (Kusunose et al., 2016) and the evidence that OSA is associated independently with decreasing left ventricular systolic function and reduced right ventricular function (Korcarz et al., 2016). Nevertheless, this cardiac remodeling in OSA patients –individuals characterized by sustained systemic acidosis, hypercapnia and cerebral vasodilation– might not be present during intermittent hypoxia exposure in healthy subjects, in which alkalosis and hypocapnia, both induced by the hyperventilation caused by adrenergic drive, are evident and probably lead to cerebral vascular constriction and reduced effects of hypoxic insult (Viscor et al., 2014). Consequently, some of the changes and/or adaptive responses found in these pathological conditions must be interpreted with caution. In conclusion, there is no solid evidence for pernicious cardiac remodeling, but rather the opposite, after intermittent hypoxia in healthy individuals, whether accompanied by physical exercise or not. Table 3 contrasts the deteriorated cardiac function in OSA patients in comparison to several studies demonstrating, both in experimental animal models and human coronary patients, the positive effects of intermittent hypoxia exposure on cardiac function.

**Table 3:** Examples of the positive effects of intermittent hypoxia exposure on cardiac pathologies.

**Subjects**	**Time of hypoxia**	**Hypoxia method**	**Hypoxia dosage**	**Outcome**	**References**
Acclimatized rats (4 d−12 wk)	8 h/d × 5 d/wk (12 wk)	HC	Sea level – 7,000 m	↑Pulmonary hypertension	Ostádal et al., 1989
Coronary patients (*n* = 5)	8 walks (in 4 wk)	Progressive Altitude	900–5,200 m	↑Cardiac function	Marticorena, 1993
Rats	4 h/d (3 wk)	HC	5,000 m	↓Ischemia	Asemu et al., 1999
Rats	4 or 8 h/d (3 or 6 wk)	HC	5,000 or 7,000 m	“Dose dependent” opposite effects	Asemu et al., 2000
Coronary patients (*n* = 8)	4 h/wk (13 wk)	HC	4,000 m	↑Cardiac function ↑NO	Marticorena et al., 2001
Guinea pigs	Chronic vs. sea-level	Altitude	4,500 m	↑Efficiency in generate ATP	Reynafarje and Marticorena, 2002
Rats	20 d	HC	4,500 m	↓Aging remodeling	Chouabe et al., 2004
Coronary patients (*n* = 6)	4 h/wk (14 wk)	HC	2,400–4,000 m	↑Myocardial perfusion	Valle et al., 2006
Rats	4 h/d × 5 d/wk (22 d)	HC	5,000 m	↑Myocardial capillaries	Panisello et al., 2007
Rats	5 h/d (5 wk)	HC + exercise	6,000 m	↑Cardiac function	Magalhães et al., 2013
Rats	5 h/d (5 wk)	HC + exercise	6,000 m	↑Heart mitochondrial function after DOXO treatment	Magalhães et al., 2014
OSA patients	Chronic	OSA	OSA	↓Ventricular function	Korcarz et al., 2016
OSA patients	Chronic	OSA	OSA	↓Ventricular function	Kusunose et al., 2016

*Altitude, geographic altitude; DOXO, Doxorubicin treatment; HC, Hypobaric Chamber; NO, nitric oxide; OSA, Obstructive sleep apnea*.

### Treatment of bronchial asthma

Despite modern advances in the treatments, bronchial asthma continues being a potentially severe illness. All treatments focus on the improvement of bronchial obstruction, but nowadays we do not have an etiological definitive treatment. Bronchial asthma generates great dependence on a variety of medications and therefore frequently submits the patient to derived complications (Chiu et al., 1981; Fairfax et al., 1999; van der Woude et al., 2001; Salpeter et al., 2006). The Experts Committee of the United States Food & Drug Administration published a consensus document about the risks of the antiasthma medications, some of them potentially lethal (DeNoon, 2008).

For that reason, every procedure that could diminish pharmacological dependence among asthmatic patients should be considered a benefit. IEHH programs represent a realistic possibility to apply a minimally aggressive non-pharmacological approach that would reduce bronchial obstruction and pharmacological dependence in these patients.

As early as the nineteenth century there was social wisdom and medical knowledge that respiratory illnesses may improve in the mountains. The sanatoriums for respiratory patients, traditionally, were located at moderate altitude in the mountains. Notable examples were the Dutch Asthma Center, Davos, Clavadel, at 1,686 m over the sea level (Switzerland) and the Istituto Pio XII, Misurina, Auronzo at 1,756 m (Italy) devoted to childhood bronchial asthma. The first medical reference we found about bronchial asthma and altitude is an inquiry between the doctors of Davos referring that 133 among their 143 patients with bronchial asthma that spent their holidays in this mountain town, did not present any acute episode of asthma and that 81% reported persistent improvement of the illness (Turban and Spengler, 1906).

Moreover, different epidemiological studies showed the beneficial effects of living at moderate altitude in the prevalence and severity of bronchial asthma (van Velzen et al., 1996; Yangzong et al., 2006; Droma et al., 2007; Kiechl-Kohlendorfer et al., 2007; Sy et al., 2007). However, acute hypoxia exposure, as occurs with acute altitude exposure, as in many other stress situations, can induce an asthmatic episode of bronchoconstriction. On the other hand, when the acclimatization process advances, the asthmatic illness improved or even disappeared (Allegra et al., 1995; Christie et al., 1995; Cogo et al., 1997, 2004; Gourgoulianis et al., 2001; Karagiannidis et al., 2006; Schultze-Werninghaus, 2006, 2008). Regrettably, this improvement vanished upon returning to the sea level.

The triad altitude exposure-hypoxia-acclimatization produces a number of physiological changes, some of which are accepted as related to the improvement of bronchial asthma: (a) a different breathing control pattern (Harrison et al., 2002; Serebrovskaya et al., 2003), (b) mitochondrial changes that optimize oxygen metabolism during the normal acclimatization process (Levett et al., 2012), and (c) the decrease in free radicals and the associated anti-inflammatory and immunosuppressive effects (Meehan, 1987; Simon et al., 1994; Serebrovskaya et al., 2003; Ohta et al., 2011; Oliver et al., 2013).

Since bronchial asthma improves with acclimatization to altitude and IEHH stimulates the acclimatization process (Rodríguez et al., 1999, 2000; Casas H. et al., 2000; Casas M. et al., 2000; Ibáñez et al., 2000; Ricart et al., 2000), it could be hypothesized that IEHH improves bronchial asthma. In fact, some medical studies have shown the usefulness of intermittent hypoxia exposure as a treatment for bronchial asthma (Harrison et al., 2002; Serebrovskaya et al., 2003). However, due to the different techniques and protocols used to produce the hypoxia and the wide dispersion of data, further research is required to design more effective protocols for intermittent hypoxia exposure that could prove useful in treating this disease. The ultimate objective of such treatments must be to reduce bronchial obstruction and the dependence on potentially dangerous drugs. Moreover, if protocols demonstrate a good response in bronchial asthma mitigation, they could also be useful for treating other illnesses with inflammatory backdrop. Table 4 summarizes some of the results that demonstrate a favorable effect of exposure to hypoxia on the symptoms of bronchial asthma.

**Table 4:** Examples of the application of intermittent hypoxia exposure on bronchial asthma patients.

**Subjects**	**Time of hypoxia**	**Hypoxia method**	**Hypoxia dosage**	**Outcome**	**References**
Asthmatic (*n* = 143)	1–3 mo (living at Davos)	Altitude	1,686 m	Improvement in 133 patients	Turban and Spengler, 1906
Asthmatic (*n* = 14)	5 weeks (living at Davos)	Altitude	1,686 m	Improvement in patients with HDM IgE-meditated allergy	Simon et al., 1994
Asthmatic (*n* = 11)	Mt Rosa & near Mt Everest BC (after 3–6 d trekking)	Altitude	4,559 and 5,050 m	↓Bronchial responsiveness to hypoosmolar aerosol 72 h after arrival	Allegra et al., 1995
Asthmatic (*n* = 14)	1 mo (living at Davos)	Altitude	1,686 m	↑Airway responsiveness to histamine after return to SL in children with atopic asthma at altitude	Christie et al., 1995
Asthmatic (*n* = 16)	1 mo (living at Davos)	Altitude	1,560 m	↓Airways inflammation	van Velzen et al., 1996
Asthmatic (*n* = 11)	3 d trekking from 2,800 to 5,050 m	Altitude	5,050 m	↓Bronchial response	Cogo et al., 1997
Children (*n* = 874) Epidemiologic study	Chronic	Altitude	SL – 1,200 m	↓Prevalence ↓morbidity of bronchial asthma in children at altitude	Gourgoulianis et al., 2001
Athletes (*n* = 40; 20 asthmatic)	IHT 6 × (5:5) 15 sessions in 3 wk	NH	≈6,800 m	Improvement in symptoms ↓Medication use	Harrison et al., 2002
Central Tibet epidemiologic study	Permanent stay	Altitude	3,000–4,500 m	↓Prevalence ↑Risk due to western lifestyle	Yangzong et al., 2006
Asthmatics (*n* = 11)	3 wk (living at Davos)	Altitude	1,686 m	↓local airway inflammation	Karagiannidis et al., 2006
Asmathics (*n* = 296) Retrospective review	2 wk−9 mo	Altitude	1,500–1,800 m	Beneficial effect, in particular in steroid-dependent patients	Schultze-Werninghaus, 2006
Epidemiologic ISAAC–HWO study 13–14 y old children (*n* = 3,196)	Chronic (living in Lhasa)	Altitude	3,658 m	↓Prevalence (Asthma prevalence in Lhasa was the lowest worldwide in ISAAC study)	Droma et al., 2007
Epidemiologic study Hospitalized asthmatic children 6–11 y old (*n* = 305)	Chronic	Altitude	450–1,800 m	↓Risk of hospitalization for atopic asthma	Kiechl-Kohlendorfer et al., 2007
Epidemiologic study (*n* = 9984)	Chronic	Altitude	1,500 m	↓Prevalence ↓Asthma-like symptoms	Sy et al., 2007
Asthmatics (*n* = 428) Retrospective review	3 wk−9 mo	Altitude	1,500 m	Beneficial effects beyond the effects of allergen avoidance	Schultze-Werninghaus, 2008
Mice (*in vivo*; lymphoid organs)	2 h	NH	FiO_2_ = 0.08 FiO_2_ = 0.21 FiO_2_ = 1	↓T cell activation T cell activation *in vivo* is dependent on localization and decrease with hypoxia	Ohta et al., 2011
Mountaineers (*n* = 27)	Alpine activities 11–18 d over 3,777 m	Altitude	3,777 m	↓Development of new immunity in humans	Oliver et al., 2013

*Altitude, geographic altitude; BC, Base camp; FiO_2_, Fraction of inspired oxygen; HC, Hypobaric Chamber; HDM, house dust mite; IHT, Intermittent hypoxic training alternating hypoxia with normoxia along the session; ISSAC, International Study of Asthma and Allergies in Childhood; WHO, World Health Organization*.

### Neurological impact of hypoxic exposure

Hypobaric hypoxic exposure at altitude, usually long-term, results in several pathophysiological and psychological conditions associated with the nervous system. The term high altitude deterioration (HAD) was first used by members of early Mount Everest expeditions to denote the deterioration in mental and physical condition due to prolonged time spent at high altitudes (Ward, 1954). It is well known among climbers that staying at extreme altitudes for long periods is deleterious (Milledge, 2003). Manifestations vary depending on the altitude reached and the individual's hypoxia tolerance, but include acute and chronic mountain sickness, memory loss and high-altitude cerebral edema (Lieberman et al., 1994; Hornbein, 2001; West et al., 2013). Acute mountain sickness generally occurs 6 to 12 h after an unacclimatized person ascends to 2,500 m or higher (Bärtsch and Swenson, 2013). As a result, cognitive function may be impaired under hypoxia (Virués-Ortega et al., 2006), although the physiological changes that occur during acclimatization prevent mountain sickness. Given the acclimatization-like responses triggered by intermittent hypoxic exposure, it offers protection against severe hypoxia exposure damage and has been reported to produce beneficial effects (Kushwah et al., 2016). Our group reported how short-term (3-h sessions on three consecutive days) IEHH with surface muscle electrostimulation increased the concentration of circulating progenitor cells in the peripheral blood of humans (Viscor et al., 2009). However, we were unable to reproduce these results later in healthy patients and those with traumatic brain injuries (Corral et al., 2014a,b), thus raising doubts about the potential role of hypoxia exposure in the release of stem cells to circulation and its involvement in the tissue regeneration process. In any case, the translation of the physiological effects of IEHH to humans is not straightforward in the field of neurology.

The brain's protective mechanisms involved in intermittent exposure to hypoxia have been widely studied using experimental animal models, and numerous beneficial effects have been reported. Intermittent hypoxia facilitates the proliferation of neural stem cells *in situ* in the subventricular zone and dentate gyrus of rat brains (Zhu et al., 2005; Ross et al., 2012). Xu et al. (2007) described a time-dependent migration of neural progenitor cells (NPC), promoted by hypoxia-induced astrocytes, thereby suggesting a role for astrocytes in NPC replacement therapy in the central nervous system. Intermittent hypoxia stimulated hippocampal angiogenesis and neurogenesis and improved short-term memory indices in control mice; and, in brain-injured mice, it reduced injury size and prevented memory impairments (Bouslama et al., 2015). It was recently reported that activation of HIF-1 is involved in hyperglycemia-aggravated blood-brain barrier disruption in an ischemic stroke model (Zhang et al., 2016b). Moreover, glycemic control by insulin abolished HIF-1α upregulation in diabetic animals and reduced blood-brain barrier permeability and brain infarction (Zhang et al., 2016b). Acute intermittent hypoxia can trigger spinal plasticity associated with sustained increases in respiratory, somatic and/or autonomic motor output (Streeter et al., 2017).

In rats, intermittent hypobaric hypoxia preconditioning caused a reduction in the degree of brain injury following ischemia-reperfusion by reducing hippocampal neuronal apoptosis by local upregulation of neuroglobin and Bcl-2 expression (Wu et al., 2015). Neuroglobin is an intracellular monomer hemoprotein that was discovered by Burmester et al. (2000) and is expressed in the central and peripheral nervous system, cerebrospinal fluid, retina and some endocrine areas of the brain (Burmester and Hankeln, 2004). It reversibly binds oxygen with a higher affinity than normal adult hemoglobin, and plays a critical role in brain tissue protection facing a possible oxygen delivery shortage (Ascenzi et al., 2016). Bcl-2 is an anti-apoptotic protein localized in the outer membrane of the mitochondria; overexpression of Bcl-2 in neurons can inhibit neuron apoptosis induced by ischemia-reperfusion injury by maintaining the integrity of mitochondria (Xing et al., 2008; Zhang et al., 2008).

Kushwah et al. (2016) also explored the ameliorating potential of intermittent hypoxia against the detrimental effects of unpredictable chronic mild stress (UCMS) on anxiety and depression-like behavior in rats, through the enhancement of neurogenesis in the hippocampus, a response mediated by brain derived neurotrophic factor (BDNF). In the postischemic rat brain, intermittent hypoxia intervention rescued ischemia-induced spatial learning and memory impairment by inducing hippocampal neurogenesis and functional synaptogenesis via BDNF expression (Tsai et al., 2013).

Nowadays, intermittent hypoxia exposure is known to enhance neurogenesis at multiple stages. Notch1 is a transcription factor in the neuron's membrane that regulates several stages of neurogenesis and promotes differentiation of progenitor cells into astroglia. Notch1 is activated by hypoxia *in vivo*, and such activation has been shown to be required for hypoxia-induced neurogenesis (Zhang K. et al., 2014a). Chronic IEHH pretreatment can reduce cerebral ischemic injury, which, as similarly reported for myocardium (see above), is mediated through upregulation of the expression and activity of mitochondrial membrane ATP-sensitive potassium channel (mitoKATP) (Zhang et al., 2016a). As is well known, hypoxia inducible factor-1 (HIF-1) is the key transcription factor that controls early adaptive responses to the lack of oxygen in mammalian cells. HIF-1α and HIF-1β expression was measured during acclimatization to hypobaric hypoxia in the rat cerebral cortex, and neurons, astrocytes, ependymal cells and possibly endothelial cells were the cell types that expressed HIF-1α (Chávez et al., 2000). Thus, the vascular remodeling and metabolic changes triggered during prolonged hypoxia may restore normal oxygen delivery levels to brain tissue (Agani et al., 2002; Chavez and LaManna, 2002).

Finally, there is solid evidence of the beneficial effects of intermittent hypoxia exposure on spinal cord neural tissue repair. Complete or incomplete spinal cord injuries are characterized by spared synaptic pathways below the level of the injury. Intermittent hypoxia elicits plasticity in the spinal cord and strengthens these spared synaptic pathways, expressed as respiratory and somatic functional recovery in both experimental animals and humans with traumatic spinal cord injury (Navarrete-Opazo et al., 2015, 2017a,b; Dougherty et al., 2017; Trumbower et al., 2017). Table 5 lists studies reporting beneficial neurological impact after a wide range of intermittent hypoxia exposure protocols.

**Table 5:** Examples of the effects of intermittent hypoxia exposure with favorable neurological impact.

**Subjects**	**Time of hypoxia**	**Hypoxia method**	**Hypoxia dosage**	**Outcome**	**References**
Rats	6 h, 12 h, or 1, 4, 7, 14, or 21 d	HC	BP = 380 Torr (≈5,500 m)	↑Hct ↑GLUT-1 ↑VEGF ↑brain HIF-1α until 14 d ↓brain HIF-1α at 21 d	Chávez et al., 2000
Cell culture	4 h	NH	1%O _2_ + 5%CO_2_ + N_2_	↑HIF-1α (NO interferes expression)	Agani et al., 2002
Rats (male)	11–13 min	Ischemia	Ischemia after cardiac arrest	↑HIF-1α 12 h−7 d ↑IGF-1	Chavez and LaManna, 2002
Rats (male) (3 groups)	4 h/d for 2 wk	HC	3,000 m 5,000 m Control normoxic	↑BrdU-labeled cells in SVZ and DG (NPC) in rat brain	Zhu et al., 2005
Astrocytes and NPC culture from brain cortex of newborn rats	6, 12, 18, and 24 h	NH	1%O_2_ + 5%CO_2_ + N_2_ (astrocytes)	↑Migration of NPC by hypoxia-induced astrocytes (maximal at 18 h)	Xu et al., 2007
Neuronal cultures of 16–18 days old fetuses of Sprague–Dawley rats	6 h “ischemia” 48 h “reperfusion”	NH	Anoxic atmosphere (5%CO_2_ + 95%N_2_)	Mitochondrial dysfunction & ER stress ⇒ neuronal apoptosis ↑Bcl-2 ⇒↓Apoptosis	Zhang et al., 2008
Rats (*n* = 122) -Ischemia MCA *n* = 42 -Ischemia MCA + post-cond *n* = 42 -Control group *n* = 40	60 min MCA ischemia post-cond (60 min after reperfusion): reperfusion for 30 s, MCA occluded for 5 cycles × 30 s	Ischemia (MCA occlusion)	Unknown (ischemia)	↑Bcl-2 ↑Hsp70 ↓Cytochrome *c* ↓Bax translocation to the mitochondria ↓Caspase-3 ↓Infarct volume ↓Oxidative stress ↑Neurologic scores	Xing et al., 2008
Neonatal mice: acute IH and control group	40 min 20 × (1:1)	NH	FiO_2_ = 0.10 (10% O_2_)	↑SVZ derived NPC *in vitro*	Ross et al., 2012
Rats (*n* = 55) Groups: with or without MCAO and/or IH, and/or zidovudine	4 h/d for 7 d	NH	FiO_2_ = 0.12 (12% O_2_)	Post brain ischemia: ↑Synaptogenesis via BDNF ↑Neurogenesis ↑Spatial learning and memory	Tsai et al., 2013
TBI medical history human males. 4 groups: -Exercise and SES (*n* = 5) -Cycling (*n* = 5) -IHH and SES (*n* = 6) -Control (*n* = 5)	2 h/d × 3 d/wk for 12 wk	HC	4,500 m	↑CPC No changes in psychological tests ↑Aerobic capacity or workload	Corral et al., 2014a
Mice (wildtype vs. Notch1 KO)	4 h/d during consecutive 28 d	HC	2,000 m	↑Notch1 ↑Hypoxia induced neurogenesis	Zhang K. et al., 2014a
Newborn mice with brain injury 3 Groups (*n* = 373) -Hypoxia separated from the mother -Normoxia separated from the mother Control with mother	20 events/h, 6 h/d from postnatal day 6 (P6) to P10	NH	FiO_2_ = 0.08 (8% O_2_)	Control mice: ↑Hippocampal angiogenesis ↑Neurogenesis ↑Short-term memory indices Brain-injured mice: ↓Injury size ↓Memory impairments	Bouslama et al., 2015
Rats (*n* = 48) hypocampal CA1 region. 4 groups with or without ischemia-reperfusion and IHH	Hypoxia for 4 d once a day I/R 8 min	*In vivo* I/R	Unknown (ischemia)	↑Surviving cells in the hippocampal CA1 in IHH+IR ↑Bcl-2	Wu et al., 2015
Rats with C2 medular hemisection (*n* = 32)	IHT 10 × (5:5) intervals (total 95 min) for 7 d	NH	FiO_2_ = 0.105 (10.5% O_2_)	↑Breathing capacity ↑Contralateral diaphragm (adenosine dependent) −2° intercostal muscle (adenosine independent)	Navarrete-Opazo et al., 2015
Rats (male) with Chronic Mild Stress induced depression (*n* = 60) and controls (*n* = 20)	4 h/d for 2 wk	HC	5,000 m	Avoid neuronal loss ↑Neurogenesis ↑BDNF–TrkB signaling	Kushwah et al., 2016
Rats (*n* = 195) 6 groups with chronic 5 wk stress and/or IHH or IMIP or antagonist of mitoKATP	6 h/d for 28 days	HC	5,000 m	↑Expression and activity of mitoK_ATP_ ↓Cerebral ischemia injury ↓UCMS	Zhang et al., 2016a
Rats with C2 medular hemisection (*n* = 27) with or without hypoxia + adenosine inhibitor	8 wk post-lesion 5 min hypoxia, 5-min normoxic 10 times (95 min) 7 d, AIH 3/wk 8 wk	NH	FiO_2_ = 0.105 (10.5% O_2_)	↑Tidal volume and bilateral diaphragm activity (enhanced by adenosine receptor inhibitor) for 4 wk	Navarrete-Opazo et al., 2017b
Rats with spinal C2 hemisection. 1) 7d after lesion 2) 7wk after lesion + serotonin receptor antagonist	IHT 10 × (5:5) intervals (total 110 min)	NH	FiO_2_ = 0.105 (10.5% O_2_)	↑Breathing capacity Serotonin independent in acute (2wk) and serotonin dependent in chronic (8wk)	Dougherty et al., 2017
Humans incomplete spinal cord injured (*n* = 35): IH + BWSTT (*n* = 18) NX + BWSTT (*n* = 17)	IHT 15 × (1.5:1.5) intervals for 5 consecutive d + 3 d/wk for 3 wk	NH	FiO_2_ = 0.09 (9% O_2_)	↑Walking recovery and endurance (up to 5wk)	Navarrete-Opazo et al., 2017a
Rats (*n* = 12)	IHT 3 × (5:5)	NH	FiO_2_ = 0.11 (11% O_2_) alternating with hyperoxia FiO_2_ = 0.5 (O_2_ 50%)	↑or↓in firing rate of midcervical interneurons altering connectivity	Streeter et al., 2017
Men with chronic incomplete spinal cord injury (*n* = 6) (double-blind, crossover study)	IHT 15 × (1.5:1) intervals for 5 consecutive d + hand opening practice	NH	FiO_2_ = 0.09 (9% O_2_)	↑Hand dexterity, function, or opening in all participants	Trumbower et al., 2017

*In IHT protocols hypoxia was alternated with room air (FiO_2_ = 0.209) if nothing else is indicated. AIH, acute intermittent hypoxia; BDNF, brain derived neurotrophic factor; Bcl-2, B cell lymphoma/leukemia-2; BP, barometric pressure; BrdU, 5-Bromo-2-deoxyuridine-5-monophosphate; BWSTT, body weight-supported treadmill training; CAO, carotid artery occlusion; CIHH, chronic intermittent hypobaric hypoxia; CPC, circulating progenitor cells; DG, dentate gyrus; FiO_2_, Fraction of inspired oxygen; GLUT-1, glucose transporter-1; HC, Hypobaric Chamber; Hct, hematocrit; HIF, Hypoxia inducible factor; Hsp70, heat shock protein70; IGF-1, insulin-like growth factor-1; IH, intermittent hypoxia; IHH, intermittent hypobaric hypoxia; IHT, intervallic hypoxic training alternating hypoxia and normoxia along the session; IMIP, imipramine; I/R, ischemia-reperfusion; KO, knockout mutant; MCA, medium cerebral artery; MCAO, middle cerebral artery occlusion; NH, normobaric hypoxia; NO, nitric oxide; NPC, neural progenitor cells; NX, normoxia (placebo); ER, endoplasmic reticulum; TBI, traumatic brain injury; UCMS, Unpredictable Chronic Mild Stress; VEGF, vascular endothelial growth factor; SES, surface electrical stimulation; SVZ, subventricular zone*.

### Other pathological conditions where the use of intermittent hypoxia exposure has potential therapeutic value

Since the altitude-hypoxia-acclimatization triad is known to have some antioxidant, anti-inflammatory and immunosuppressive effects (Meehan, 1987; Meehan et al., 1988; Ohta et al., 2011; Oliver et al., 2013), a benefit in some other pathologies related to immune response, such as psoriasis, atopy, arthritis or autoimmune pneumonitis can be expected. This is still a controversial field with no extensive clinical studies available, although some medical descriptive studies point to potential future research opportunities (Singh et al., 1977; Vocks et al., 1999; Engst and Vocks, 2000; Steiner, 2009).

Intermittent exposure to both normobaric and hypobaric hypoxia has been related to some protective effects (Cai et al., 2003; Costa et al., 2013) and beneficial outcomes in several pathological conditions, especially in those related to metabolic syndrome (Marquez et al., 2013; Leone and Lalande, 2017; Serebrovska et al., 2017). The possible role of intermittent hypoxia on body weight control has also attracted considerable attention. In addition to improving exercise performance and diet control, intermittent exposure protocols to normobaric and hypobaric hypoxia have been applied in an attempt to potentiate weight loss, showing in some cases positive short-term results (Haufe et al., 2008; Netzer et al., 2008; Lippl et al., 2010; Wiesner et al., 2010; Cabrera-Aguilera et al., in press). However, a long-term study failed to demonstrate permanent body weight reduction after IEHH (Gatterer et al., 2015), suggesting that additional research is needed to clarify the discrepancies reported in this field.

In summary, recent reports call for increased attention to the potential benefits of the application of intermittent hypoxia protocols in several clinical areas (Dale et al., 2014; Mateika et al., 2015). It is likely that future studies will yield important new information regarding potential therapeutic uses of intermittent hypoxia. Table 6 lists a non-exhaustive but representative sample of studies reporting favorable impact of intermittent hypoxia exposure in other pathological conditions.

**Table 6:** Examples of the effects of intermittent hypoxia exposure with favorable impact in other pathological conditions.

**Subjects**	**Time of hypoxia**	**Hypoxia method**	**Hypoxia dosage**	**Outcome**	**References**
Epidemiological study (1965–1972) in 20,000 altitude native men vs. to 130,700 lowlanders (760 m)	Chronic	Altitude	3,692–5,538 m	↓Diseases ↓Morbidity rates	Singh et al., 1977
Rats (*n* = 24)	8 h/d for 30 d	HC	5,000 m	↑Resistance to epileptogenic action of penicillin	Agadzhanyan and Torshin, 1986
Healthy men (*n* = 7) (operation Everest II)	40 d	HC	7,620 m	↓T cell function ↓PHA-stimulated thymidine uptake	Meehan, 1987
Healthy men (*n* = 8) (operation Everest II)	4 weeks 5 d at 2,286m 28 d at 7,620 m (gradually) peaks 1–4 h at 8,839 m	HC	2,286 m 7,620 m 8,839 m	↓Phytohemagglutinin-stimulated thymidine uptake and protein synthesis in mononuclear cells ↑Monocytes ↑Plasma IgM & IgA ↓T-cell activation	Meehan et al., 1988
Essential hypertension patients	30 min/d, 5 d/wk for 3 wks	HC	3,500 m	↓BP ↓Blood vol ↓[Na]serum ↑Microcirculation ↑PO_2_ tissue ↓cholesterol	Aleshin et al., 1993
Tibetan natives at moderate (M) and high (H) altitudes	Chronic	Altitude	M: 2,000–3,000 m H: 4,000–4,700 m	↓HR ↓HVR ↓V_E_max in group at H altitude	Ge et al., 1994
Psoriasis affected patients (*n* = 76)	4 wk	Altitude	1,560 m	↓Psoriasis no changes in plasma cortisol	Vocks et al., 1999
Neurodermitis patients (*n* = 31,438)	4 wk	Altitude	1,560 m	↓Dermatosis ↓Psoriasis ↓ECP	Engst and Vocks, 2000
Coronary patients (*n* = 46)	22 sessions 3 h/d	HC	3,500 m	↓Total cholesterol ↑HDL ↓LDL ↓VLDL ↓TG	Tin'kov and Aksenov, 2002
Mice (*n* = 9)	IHT 5 × (6:6)	NH	FiO_2_ = 0.06	↑EPO; ↑heart HIF-1α	Cai et al., 2003
Obese subjects (BMI > 27) (*n* = 20) -Hypoxia (*n* = 10; 8 w + 2 m) -Sham (*n* = 10; 8 w + 2 m)	1.5 h/d, 3 d/wk for 8 wk exercising at 60% VO_2_max	NH	FiO_2_ = 0.15 (≈2,500 m) FiO_2_ = 0.201 (≈450 m)	↓BMI and ↑BW loss ↓cholesterol ↓TG and ↓LDL	Netzer et al., 2008
Healthy men (*n* = 20) -Hypoxia (*n* = 10) -Normoxia (*n* = 10)	1 h/d, 3 d/wk for 4 wk exercising at 3 mmol/L Lac HR	NH	FiO_2_ = 0.15 (≈2,760 m) FiO_2_ = 0.21 (≈SL)	↓Body fat content ↓TG ↓HOMA-Index fasting insulin and ↓AUCins	Haufe et al., 2008
Atopic dermatitis and psoriasis patients (mini-review)	12 d−4 wk	Altitude	1,560 m	↓Symptoms	Steiner, 2009
Obese men (*n* = 20)	1 week	Altitude	2,650 m	↓BW ↑BMR ↓Food intake ↑Basal leptin ↓diastolic BP	Lippl et al., 2010
Overweight to obese subjects (*n* = 24)	4 wk training under hypoxia at 65% VO_2_max	NH	FiO_2_ = 0.15 (≈2,760 m)	↑Physical fitness ↓Metabolic risk markers ↑Body composition	Wiesner et al., 2010
Mice (*in vivo*; lymphoid organs)	Acute 2 h exposure	NH	FiO_2_ = 0.08 FiO_2_ = 0.21 FiO_2_ = 1 (variable in organs: thymus < lymphoid nodes < spleen)	↑T-cell activation in better oxygenated tissues. T-cell activation *in vivo* is dependent on localization and decrease with hypoxia	Ohta et al., 2011
Mice (WT vs. KO)	Myoblast cell culture	NH	5 vs. 21%	Endogenous EPO promotes satellite activation and functional recovery after muscle injury	Jia et al., 2012
Rats (*n* = 8)	3 h/d for 6 d	HC	5,500 m	Injured excitotoxic brain: ↑EPO ↓Lipid peroxidation ↓Apoptotic cell death	Costa et al., 2013
Healthy adult sedentary men (*n* = 28)	2 × 20 min/d × 3 d/wk for 10 weeks	HC (CVAC) 5 fluctuations/min	Progressive: SL - 3,048 m (wk 1) SL - 6,096 m (wk 5–10)	No changes in Hct, [Hb], cholesterol and insulin ↓Fasting plasma glucose ↓Plasma glucose in response to oral glucose tolerance test	Marquez et al., 2013
Recreationally active mountaineers (*n* = 10; 3 w + 7 m)	28 h	Altitude	3,777 m	↓Immune response	Oliver et al., 2013
Obese patients (BMI > 30 kg/m^2^) (*n* = 16; 4 w + 12 m)	52 sess of 90 min (8 mo)	NH	Exercise: 3,500 m Rest: 4,500 m	No added effects by hypoxia to those provoked by moderate intensity exercise	Gatterer et al., 2015
Prediabetic adult patients (*n* = 11; 6 w + 5 m)	3 sess/wk for 3 wk	NH (IHT 5:5)	FiO_2_ = 0.12 (≈4,400 m)	↑mRNA expression of HIF-1α and target genes ↓Fasting plasma glucose ↓Plasma glucose response to 2 h post-oral glucose tolerance test	Serebrovska et al., 2017
Trained rats (*n* = 78) -Basal (*n* = 6) -Hypoxia (*n* = 24) -Hypoxia + LAE (*n* = 24) -Normoxia (*n* = 24)	4 h/d for 14 d	HC	4,000 m	m. soleus: ↑Histological muscle damage recovery No change in fiber types ↓Fiber size ↑Capillarisation ↑VEGF expression ↑Citrate synthase activity ↑PGC-1α	Rizo-Roca et al., 2017a
Trained Rats (*n* = 78) -Basal (*n* = 6) -Hypoxia (*n* = 24) -Hypoxia + LAE (*n* = 24) -Normoxia (*n* = 24)	4 h/d for 14 d	HC	4,000 m	m. soleus: ↑Mitochondrial biogenesis markers ↑Mitochondrial dynamics markers ↓Oxidative stress ↓Apoptotic signalling	Rizo-Roca et al., 2017b
Rats (*n* = 28) -Normoxia sedentary (*n* = 7) -Normoxia + EET (*n* = 7) -Hypoxia sedentary (*n* = 7) -Hypoxia + EET (*n* = 7)	5 h/d for 4 wk	HC	6,000 m	↓Food intake ↓Body weight gain ↓Oxygen consumption Additive effect of IHH+EET	Cabrera-Aguilera et al., in press

*3 mmol/L Lac HR, heart rate corresponding to the 3 mmol/L lactate value in the FiO_2_-specific incremental test; Altitude, geographic altitude; AMS, acute mountain sickness; AUCins, Insulin response (area under curve) to oral glucose tolerance test; BMI, body mass index; BMR, basal metabolic rate; BP, arterial blood pressure; BW; body weight; CVAC, Cyclic Variations in Altitude Conditioning; ECP, eosinophil cationic protein; EET, endurance exercise training; EPO, Erythropietin; FiO_2_, Fraction of inspired oxygen; [Hb], Blood hemoglobin concentration; HC, Hypobaric Chamber; Hct, Hematocrit; HDL, high density lipoproteins; IHT, Intermittent hypoxic training alternating hypoxia with normoxia along the exposure protocol; HOMA-Index, homeostatic model assessment index of insulin resistance; HR, Heart rate; HVR, hypoxic ventilatory response; IHH, intermittent hypobaric hypoxia; IHT (6:6), intermittent hypoxic training, alternating 6 min hypoxia with 6 min normoxia along the session; LAE, light aerobic exercise; LDL, low density lipoproteins; NH, normobaric hypoxia; TG, plasma triglycerides; V_E_max; maximal exercise pulmonary ventilation; VLDL, very low density lipoproteins*.

## Intermittent hypoxia in sport

The benefits of intermittent hypoxia programs in competitive sport are still a subject of scientific debate. Table 7 shows several examples of intermittent hypoxia exposure effect on the improvement of human physical performance. A prior consideration to bear in mind when dealing with this topic is that two inherent factors justify the diversity of results in the field of elite sport: (a) the narrow margin of improvement detectable in elite athletes; and (b) the limitation in the sample size when performing studies with this population. Both factors contribute to reduced statistical power in most of these studies.

**Table 7:** Examples of the intermittent hypoxia exposure on the improvement of human physical performance.

**Subjects**	**Time of hypoxia**	**Hypoxia method**	**Hypoxia dosage**	**Outcome**	**References**
Elite alpinists (*n* = 5; 1 w + 4 m)	1 wk chronic + 38 h for 4 d	Altitude + HC	4,350–4,807 m (1 wk at Mt Blanc) 5,000–8,500 m (4 d at HC)	↑SaO_2_ during HT ↑Altitude acclimatization	Richalet et al., 1992
Competitive (*n* = 39, 12 w + 27 m)	4 wk	Altitude	LH-TL 1,250/2,500 m	↑VO_2_max	Levine and Stray-Gundersen, 1997
Competitive (*n* = 39; 12 w + 27 m)	14 d	LH-TL LH-TH	1,200–1,400 m/2,500–3,000 m	↑VO_2_max ↑VO_2_max	Chapman et al., 1998
Alpinists (*n* = 17)	3–5 h/d for 9 d	HC	4,000–5,500 m	Vo_2_max ↑Lact/Vel	Rodríguez et al., 1999
Competitive (*n* = 126, 37 w + 89 m)	4 wk	Altitude	LH-TL 1,250/2,500 m	↑Vo_2_max in responders	Stray-Gundersen and Levine, 1999
Alpinists (*n* = 9)	2 h/d 14 d	HC	5,000 m	↑V_E_ ↑SaO_2_ during exercise at 5,000m	Ricart et al., 2000
Elite athletes (*n* = 18; 2 w + 16 m) -Hypoxia (*n* = 8; 2 w + 6 m) -Control (*n* = 10; 10 m)	10 d	NH (LH-TL)	FiO_2_ = 0.158 (≈2,200 m)	↓400-m race time ↑resting blood pH	Nummela and Rusko, 2000
Elite alpinists (*n* = 6)	3–5 h/d for 17 d	HC	4,000–5,500 m	↑TtE ↑Lact/Vel	Casas M. et al., 2000
Competitive (*n* = 23; 11 w + 12 m) -Hypoxia (*n* = 11) -Normoxia (*n* = 12)	3 h/d, 5 d/wk for 4 wk	HC	4,000–5,500 m	Marginal ↑VO_2_max (*p* < 0.07) but only in swimmers	Rodríguez et al., 2007
Elite athletes (*n* = 41) -XC Skiers (*n* = 11) -Swimmers (*n* = 18) -Runners (*n* = 12)	18 nights 13 nights 18 nights	NH and Altitude (LH-TL)	1,200m/ /2,500–3,500 m /2,500–3,000 m /2,500–3,000 m	↑VO_2_max ↑VO_2_max ↑VO_2_max	Richalet and Gore, 2008
Competitive (*n* = 28; 11 w + 17 m)	3 h/d, 5 d/wk for 4 wk	HC	4,000–5,500 m	No changes in submaximal economy	Truijens et al., 2008
Meta-Analysis of 51 studies on elite and sub-elite athletes	Diverse	Natural vs. artificial altitude (no discrimination between artificial NH or HH)	Diverse	↑Maximal endurance power output after natural and brief intermittent artificial LH-TL	Bonetti and Hopkins, 2009
Randomized, placebo-controlled, double-blind study (*n* = 40; 18 w + 22 m)	3 × 70 min/wk for 3 wk exercising + 4 × 90 min passive for 1 wk	NH	2,500 m (wk-1) 3,000 m (wk-2) 3,500 m (wk-3) 4,500 m (wk-4)	↓AMS 1st d at 3,611 m ≅AMS 2nd d at 4,559 m	Schommer et al., 2010
Healthy men (*n* = 26) Randomized, placebo-controlled, double-blind study	1 h/d for 1 wk	NH	FiO_2_ = 0.126 (≈4,500 m)	↓AMS after 8 h at FIo_2_ = 0.113 (≈5,300 m)	Wille et al., 2012
Female netballer players (*n* = 30) -Hypoxic training -Vascular occlusion training -Control	0–90° bilateral knee extension and flexion 3 sess/wk for 5 wk	NH	FiO_2_ = variable to maintain SpO_2_≈80%	↑MVC_3_ ↑MVC_30_ ↑Reps_20_	Manimmanakorn et al., 2013
USARIEM Retrospective review (*n* = 170; 37 w + 133 m)	Several	Altitude, HC and NH	4,300 m	HC or altitude much more effective than NH for ↓AMS and ↑performance during acute altitude exposure	Fulco et al., 2013
Healthy unacclimatized men (*n* = 42) -Hypoxia (*n* = 21) -Normoxia (*n* = 21)	Sleep for 14 consecutive nights	NH	FiO_2_ ≈ 0.145 (≈2,600 m)	↓AMS after 20 h at FIo_2_ = 0.12 (≈4,500 m)	Dehnert et al., 2014
Healthy men (*n* = 16) -Hypoxia (*n* = 9) -Normoxia (*n* = 7)	Resistance training 2 sess/wk for 8 wk (16 sessions in total)	NH	FiO_2_ = 0.144 (≈3,000 m)	↑Muscular endurance ↑VEGF ↑Capillary-to-fiber ratio	Kon et al., 2014
Male elite athletes (*n* = 12) -Hypoxia (*n* = 6) -Normoxia (*n* = 6)	CST 3 sess/wk for 4 wk (27 h in total)	HC	3,000 m	↑Anaerobic performance	Álvarez-Herms et al., 2015a
Elite swimmers (*n* = 54; 30 w + 24 m)	3 or 4 wk	Altitude LH-TH vs. LL-TL vs. LH-TH+TL	690/2,320 m	↓TT for LH-TH+TL	Rodríguez et al., 2015
Male trained triathletes (*n* = 18) -Hypoxia (*n* = 9) -Normoxia (*n* = 9)	1 h/d × 2 d/wk for 7 wk	NH	FiO_2_ = 0.145–0.15 (≈2,800–2,500 m)	No differences in aerobic performance	Ramos-Campo et al., 2015
Male well-trained triathletes (*n* = 16)	18 d	NH and Altitude LH-TL	1,100/2,250 m	↑VO_2_max ↓3-km run time	Saugy et al., 2016
Well-trained (*n* = 16)	Acute exposure during HIIE jump test	NH	FiO_2_ = 0.165 (≈1,900 m) FiO_2_ = 0.135 (≈3,500 m)	↑Effort perception	Álvarez-Herms et al., 2016
Well-trained men (*n* = 16)	Acute exposure during 5 × 3 45° leg press and bench press at 85% 1RM test	NH	FiO_2_ = 0.165 (≈1,900 m)	Serum ↑Lactate ↑GH	Filopoulos et al., 2017
Male endurance-trained (*n* = 15) -Hypoxia (*n* = 9) -Normoxia (*n* = 6)	IHT 6 × (5:5) at 80–85% of vVO_2_max 3 sess/wk for 6 wk	NH	FiO_2_ = 0.106 (≈5,000 m) (wk 1–2) FiO_2_ = 0.114 (≈5,500 m) (wk 3–6)	↑TtE at 95% VO_2_max	Sanchez and Borrani, 2018

*1RM, one repetition maximum; Altitude, geographical altitude; AMS, acute mountain sickness; CST, circuit strength training; FiO_2_, Fraction of inspired oxygen; HC, Hypobaric Chamber; HIIE, high intensity intervallic exercise; HT, hypoxic test (at 4,800 m equivalent attitude); IHT, Intermittent hypoxic training alternating hypoxia during exercise with normoxia during recovery between sets; Lact/Vel, Lactate/Velocity curve (up-arrow means right shift); LH-TL, Living High-Training Low; LH-TH, Living High-Training High; MVC_3_, 3 s maximal voluntary contraction; MVC_30_, 30 s maximal voluntary contraction; NH, Normobaric hypoxia; TtE, Time to exhaustion; Reps_20_, maximal number of repetitions at 20% 1RM; TT, time trial; USARIEM, United States Army Research Institute of Environmental Medicine; VO_2_max, maximal oxygen consumption capacity*.

Other sources of variability are the wide range of exposure patterns, differing hypoxic doses (or altitude), and the kind of hypoxia (hypobaric or normobaric), as is discussed below. The living high-training low (LH-TL) pattern (Levine and Stray-Gundersen, 1997) is the most widespread training schedule, although sometimes the reverse model, living low-training-high (LL-TH), is also applied, especially when artificial hypoxia is used. Moreover, studies evaluating training at altitude during permanent stays are also very usual.

In addition to other reports cited in precedent sections, a high number of studies have consistently found positive effects of IEHH programs to improve exercise performance. Thus, 4 weeks of LH-TL improved sea-level running performance in trained runners (Levine and Stray-Gundersen, 1997). Short-term intermittent hypobaric hypoxia (in a hypobaric chamber) improved the aerobic performance capacity in healthy subjects (Rodríguez et al., 1999). Intermittent hypobaric hypoxia combined with low-intensity exercise induced altitude acclimation, improved lactate threshold and ventilatory anaerobic threshold in healthy subjects (Casas M. et al., 2000). Normobaric hypoxia increased the growth hormone response to maximal resistance exercise in trained men (Filopoulos et al., 2017). Finally, a reduction in the severity of acute mountain sickness was also reported after several intermittent normobaric hypoxia protocols (Schommer et al., 2010; Wille et al., 2012; Dehnert et al., 2014).

In contrast, other studies did not detect significant improvements in exercise performance. Four weeks of IEHH did not improve oxygen transport in trained swimmers and runners (Rodríguez et al., 2007) nor did it change the submaximal economy in a group of well-trained athletes (Truijens et al., 2008). Seven weeks of normobaric hypoxia training in triathletes, caused an improvement in hematological parameters but not in the aerobic performance (Ramos-Campo et al., 2015). Finally, a recent systematic review and meta-analysis did not reveal a significant benefit of resistance training in hypoxia compared to the same training in normoxia (Ramos-Campo et al., 2018).

In general, it is commonly accepted that the application of intermittent hypoxia exposure has beneficial effects for competitive sport in the same way as for the biomedical field. As discussed above, the wide difference in effects that have been reported in the literature can be explained by individual susceptibility and the diversity of intermittent hypoxia patterns applied (Debevec and Mekljavic, 2013). The adaptive or maladaptive responses can be due to differences in the frequency, severity or duration of hypoxic episodes (Serebrovska et al., 2016). Factors such as age, sex or genotypic variability may also contribute to varying results (Almendros et al., 2014). A review of relevant publications between 1980 and 2015 concluded that evidence regarding the effects of altitude training on athletic performance is weak but that the natural stay at altitude combined with a live high-train low training strategy may provide the best protocol for enhancing endurance performance in elite and subelite athletes (Khodaee et al., 2016), thus confirming similar findings in a previous study (Bonetti and Hopkins, 2009). Finally, a recently published meta-analysis on the effect of natural or simulated altitude training in team-sport athletes conclude that hypoxic intervention appears to be a worthwhile training strategy for improvement in team-sport athletes, with enhanced performance over control groups persisting for at least 4 weeks post-intervention (Hamlin et al., 2018). Also, our recent data indicate that contractile activity seems to be necessary to trigger in skeletal muscle the adaptive responses induced by intermittent exposure to hypoxia (Rizo-Roca et al., 2018).

Although most of the expected effects of altitude training and IEHH programs have been mainly aimed at improving aerobic capacity (Stray-Gundersen and Levine, 1999; Wilber, 2004), some reports also have described benefits for strength training at moderate hypoxia levels (Nummela and Rusko, 2000; Manimmanakorn et al., 2013; Álvarez-Herms et al., 2015b, 2016). The underlying mechanism of these responses remain to be clarified, but potential psychological benefits may be derived of the perception of increased effort during hypoxic training (Álvarez-Herms et al., 2016). In addition, training during hypoxia may result in a greater increase in muscular endurance than the same training load performed in normoxia, probably because of increased angiogenesis in skeletal muscle level (Kon et al., 2014), can be involved in those improvements.

## Normobaric vs. hypobaric hypoxia: the same stimulus?

When artificial methods of producing hypoxia exposure were first developed, no attention was paid to the differences between hypobaric (low barometric pressure) and normobaric (low oxygen content in an inhaled gas mixture) hypoxia. It seems evident that the same physiological effects are expected for a determined alveolar oxygen partial pressure, regardless of the technical means used. However, data in the literature present consistent discrepancies after applying hypobaric and normobaric hypoxia, which could be attributed to alterations in other environmental parameters that affect alveolar gas composition, such as carbon dioxide partial pressure, humidity and temperature. For instance, even for the same level of oxygen partial pressure, the atmospheric composition in a small hypoxic tent with low air turnover (limited by the flow capacity of the hypoxic device) can be very different from the air composition in a hypobaric chamber. Generally, the use of powerful vacuum pumps in hypobaric chamber systems guarantees sufficient renewal of the air inside the room, thus preventing carbon dioxide accumulation and a rise in air temperature and humidity, factors that could become unbalanced in small volume hypoxic tents, especially with exercising subjects inside. In a comparative study of hypobaric (low pressure chamber) and normobaric hypoxia (hypoxic tent) during a submaximal exercise test in the same subjects, differences were observed in some cardiorespiratory and heart rate variability parameters between the two artificial hypoxia systems used (Basualto-Alarcón et al., 2012).

An interesting and dynamic debate, which is beyond the scope of this review, is currently under way among altitude researchers concerning whether or not hypobaric hypoxia induces different responses from normobaric hypoxia (Girard, 2012; Millet et al., 2012; Hauser et al., 2016; Saugy et al., 2016). Some evidences demonstrate that there are different physiological responses and outcomes between exposure to normobaric and hypobaric hypoxia conditions (Savourey et al., 2003; Fulco et al., 2013; Millet et al., 2013; Beidleman et al., 2014; Debevec and Millet, 2014; Boos et al., 2016; DiPasquale, 2017). For instance, it has been reported that the decrease in air density that accompanies the partial pressure drop of oxygen at geographic altitude affects the way in which explosive actions are executed and increases movement velocity and power during force-velocity bench presses in comparison to normobaric hypoxia (Feriche et al., 2014).

## Conclusions

A growing body of knowledge supports the beneficial effects of natural or simulated altitude techniques on health outcomes (Navarrete-Opazo and Mitchell, 2014; Millet et al., 2016; Lizamore and Hamlin, 2017). Future research should be oriented to: (1) gain more in-depth knowledge of the subcellular mechanisms involved in the hypoxic response at different tissue levels, (2) standardize hypoxia exposure methods and establish a universal method for measuring, and repeatedly applying, hypoxic dosage, (3) improve predictions of individual hypoxia tolerance to prevent possible negative consequences, (4) apply this new knowledge to the selection and education of altitude workers, (5) improve altitude acclimatization, altitude training camps and altitude competition events to benefit mountaineers, athletes and coaches, and finally (6) cautiously explore the application of IEHH in pathological conditions.

## Author contributions

GV, JV, AR, and LC contributed to the initial draft of the manuscript; GV and JT edited the document after contributions from all authors; All authors reviewed and approved the final version.

### Conflict of interest statement

The authors declare that the research was conducted in the absence of any commercial or financial relationships that could be construed as a potential conflict of interest.
